# A Free Open-Source Bayesian Vancomycin Dosing App for Adults: Design and Evaluation Study

**DOI:** 10.2196/30577

**Published:** 2022-03-30

**Authors:** Thomas Oommen, Anirudh Thommandram, Adam Palanica, Yan Fossat

**Affiliations:** 1 Pharmacy Lakeridge Health Oshawa, ON Canada; 2 Klick Applied Sciences Klick Health, Klick Inc Toronto, ON Canada

**Keywords:** medical informatics, therapeutic drug monitoring, vancomcyin, Bayesian prediction, drug monitoring, clinical data, tool development, digital health tools

## Abstract

**Background:**

It has been suggested that Bayesian dosing apps can assist in the therapeutic drug monitoring of patients receiving vancomycin. Unfortunately, Bayesian dosing tools are often unaffordable to resource-limited hospitals. Our aim was to improve vancomycin dosing in adults. We created a free and open-source dose adjustment app, VancoCalc, which uses Bayesian inference to aid clinicians in dosing and monitoring of vancomycin.

**Objective:**

The aim of this paper is to describe the design, development, usability, and evaluation of a free open-source Bayesian vancomycin dosing app, VancoCalc.

**Methods:**

The app build and model fitting process were described. Previously published pharmacokinetic models were used as priors. The ability of the app to predict vancomycin concentrations was performed using a small data set comprising of 52 patients, aged 18 years and over, who received at least 1 dose of intravenous vancomycin and had at least 2 vancomycin concentrations drawn between July 2018 and January 2021 at Lakeridge Health Corporation Ontario, Canada. With these estimated and actual concentrations, median prediction error (bias), median absolute error (accuracy), and root mean square error (precision) were calculated to evaluate the accuracy of the Bayesian estimated pharmacokinetic parameters.

**Results:**

A total of 52 unique patients’ initial vancomycin concentrations were used to predict subsequent concentration; 104 total vancomycin concentrations were assessed. The median prediction error was –0.600 ug/mL (IQR –3.06, 2.95), the median absolute error was 3.05 ug/mL (IQR 1.44, 4.50), and the root mean square error was 5.34.

**Conclusions:**

We described a free, open-source Bayesian vancomycin dosing calculator based on revisions of currently available calculators. Based on this small retrospective preliminary sample of patients, the app offers reasonable accuracy and bias, which may be used in everyday practice. By offering this free, open-source app, further prospective validation could be implemented in the near future.

## Introduction

Vancomycin is an intravenous (IV) drug that has been used for over 60 years in the treatment of Gram-positive bacterial infections. Vancomycin has a narrow therapeutic window; thus, therapeutic drug monitoring (TDM) of plasma concentrations is necessary to measure efficacy and to avoid nephrotoxicity. Traditionally, vancomycin dosing consists of giving a patient-weight–based dose, and then checking a trough concentration at an approximate steady state concentration, targeting a range between 10 and 20 mg/L [1]. Dose adjustments are often made using simple heuristics, clinical intuition, and judgement based on patient factors.

In 2020, the Infectious Diseases Society of America updated the dosing guidelines of vancomycin for severe methicillin-resistant *Staphylococcus aureus* infections. They suggest that vancomycin monitoring via an area under the curve (AUC) approach is the safest and most effective approach to TDM [1]. AUC measurement can be calculated by taking blood concentrations and performing pharmacokinetic (PK) calculations to derive an AUC estimate. This is measured by either the Sawchuk-Zaske method (ie, based on individually calculated PK parameters) or using a Bayesian inference approach (ie, a method of statistical inference using the Bayes theorem to update the probability as more data become available) [2]. According to the Infectious Diseases Society of America, Bayesian-based tools are the preferred approach to vancomycin TDM [1]. Controversy still exists regarding the adoption of AUC-only monitoring of vancomycin [3-5]. Some suggest that adopting this approach may be premature [3], and AUC dosing if carried out by 2-level approach may increase the patient burden by increasing blood draws. There is still some uncertainty on the ideal target AUC or trough level [4].

Calls for individualized Bayesian dosing tools have existed for years [6-10]; however, Bayesian TDM has failed to garner widespread adoption for many reasons [7]. The Bayesian TDM approach can require paid computer software programs. These programs typically require multiyear licensing agreements and can be unaffordable to hospital systems. These software packages often use publicly available PK models [11-14] and Bayesian algorithms to derive dosing suggestions. Most apps do not make their code public due to proprietary coding and financial interest. However, it may be assumed that the PK models and Bayesian methods are not proprietary. These Bayesian TDM apps make use of these PK models as a priori to assist in predicting individual patient PK parameters. The apps conduct mathematical calculations seamlessly without requiring the user to have a knowledge of statistics. The Bayesian inference algorithms were once limited by computing power; however, with advances in technology, they can be performed with any basic software browser via HTML and JavaScript.

As a solution to these paid software programs, we developed a proof-of-concept, free online Bayesian vancomycin dosing app, VancoCalc [15]. VancoCalc allows vancomycin TDM using trough or AUC-based targets. The app uses published PK models and user-inputted patient data as Bayesian priors to estimate vancomycin concentrations and AUC. The app requires no statistical training and is aimed to be user-friendly to assist in the implementation of Bayesian inference in vancomycin TDM. No user data are saved as all calculations are computed locally on the user’s device.

**Figure 1:**
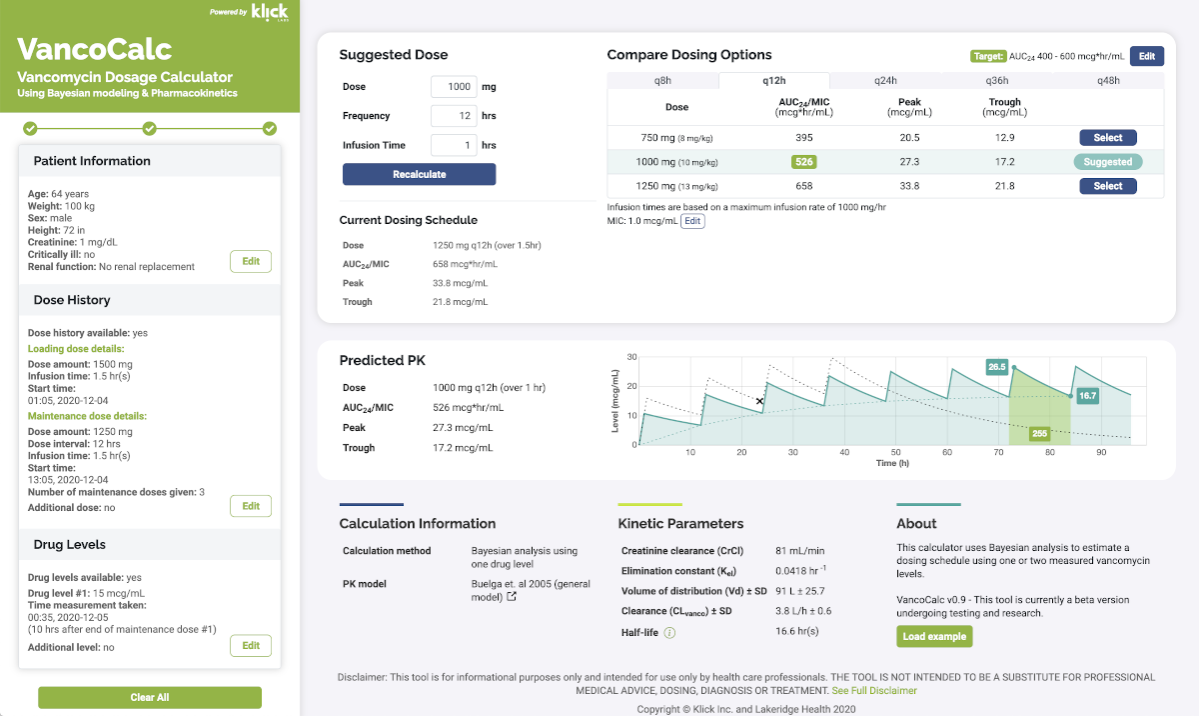
Example of the app's graphical user interface. AUC: area under the curve; MIC: minimum inhibitory concentration; PK: pharmacokinetic.

Inspiration for our new calculator was fostered from anecdotal user experience with DosOpt [16], ClinCalc [17], and BestDose [18], in hopes of making a more user-friendly app. VancoCalc adds the ability to customize target trough or AUC, permitting 2 different dose or time intervals (plus loading dose) relating to the vancomycin concentration, and the ability to explore various dosing regimens through visualization of the dose and time curve. These updates assist the clinician in assessing the impact a dosing regimen may have (Figure 1). After inputting “Bayesian Vancomycin Calculator” in a search engine, it may appear that there are many Bayesian dosing apps. However, many of these calculators use 2-level trapezoidal calculations and are not truly using Bayesian approaches.

The app’s ability to predict a patient’s vancomycin concentration (pharmacokinetic parameters) was evaluated using a data set composed of adult patients who received vancomycin at Lakeridge Health. This research aims to describe the development, design, and evaluation of a Bayesian vancomycin dosing app.

## Methods

### Development of VancoCalc App

We implemented the app as a static web app using HTML5, JavaScript, CSS, and several open-source projects. The user interface was developed using the jQuery [19], Bootstrap [20]. And Chart.js [21] frameworks. The app uses a custom library for applying Bayesian algorithms that is based on bayes.js [22] and the simple statistics [23] libraries. Markov chain Monte Carlo sampling and model fitting are carried out in the user’s browser with no data processing needed from a server or the cloud.

### App Design Bayesian Pharmacokinetic Modelling and Fitting

Bayesian dosing strategies employ population models that relate PK parameters, including volume of distribution (VD), clearance (Cl), and creatinine clearance to patient data, including age, weight, gender, serum creatinine, vancomycin dose value, and number of doses given. The Bayesian approach involves the notion of incorporating both a population PK model and measured drug concentrations from the patient to better estimate PK parameters for the individual.

The app selects a population model based on the inputted patient data. If a patient is critically ill and has a BMI of over 30 kg/m^2^ and body weight of over 100 kg, the Masich et al [14] model is used; if a patient is critically ill but is not meeting the obese criteria, the Roberts et al [13] model is used; if not critically ill but has a BMI of over 40 kg/m^2^ and body weight of over 120 kg, then the Adane et al [11] model is used; if not critically ill and BMI is less than 40 kg/m^2^, the Buelga et al [12] general population model is used. The critically ill model parameter is possible to override. Estimates of the PK parameters (VD and Cl) from the population model, adjusted for patient characteristics, form the starting point of the fitting process. The starting point begins with the mean VD and Cl of the PK model selected. The values are sampled from the parameter space, and first-order PK equations are used to calculate an expected serum concentration, which is compared to the measured concentration. This sampling and traversing of the parameter space are carried out iteratively while taking into consideration the variability of the population parameters and the variability of the serum concentration measurement. By doing this, the modelling process estimates the PK parameters that will be most consistent with drug concentrations predicted by both the population model and the measured drug concentrations.

Complex dose history can be entered involving multiple doses of varying amounts, frequencies, and schedules. Concentrations are not restricted to steady state and can be entered for any infusion and are not limited to trough levels. The calculator’s inputs and outputs are shown on the same page, side-by-side, and any modifications to the input are immediately reflected in the calculated output dynamically. The design provides a suggested dose and alternatives, but also encourages exploration and investigation for the patient cases that require it.

### App Evaluation

A retrospective observational data set was used to evaluate the app. Information was gathered via electronic chart review (Meditech) and SQL query of Antimicrobial Stewardship data repository.

The data were collected between July 2018 and January 2021 at Lakeridge Health Corporation sites in Oshawa, Bowmanville, or Port Perry, Ontario, Canada. This is a community hospital system with approximately 800 beds located in the Durham region of Ontario. Inclusion criteria were inpatients, aged 18 years and over, who received at least 1 dose of IV vancomycin and had at least 2 vancomycin concentrations drawn in relation to the vancomycin dose. Patient’s sex, age, height, weight, serum creatinine at time of vancomycin, ward, vancomycin dosing history, and vancomycin concentrations were collected. Exclusion criteria were patients who were receiving hemodialysis or continuous renal replacement while on vancomycin, or missing any data as stated in the inclusion criteria.

The first single known vancomycin concentration and matching patient variables were entered into the app. This allowed the app to estimate the patient’s individual PK parameters. Subsequent vancomycin concentrations (if available) were not inputted as the app currently only allows 2 vancomycin concentrations to be inputted. This produced a vancomycin plasma concentration time plot where the estimated second vancomycin concentration was compared to the actual concentration.

### Ethics Approval

The Research Ethics Board (REB) at Lakeridge Health approved the study (Approval 2020-13) in September 2020, Oshawa, Ontario.

### Statistical Analysis and Predictive Performance

Performance of Bayesian prediction of the second vancomycin concentration was evaluated with the median prediction error (MedPE), median absolute error (MedAE), and root mean squared error (RMSE). MedPE, MedAE, and RMSE were calculated according to Equations 1, 2, and 3, and were derived from the study by Sheiner and Beal [24]. MedPE was calculated as an index of bias, MedAE as an index of accuracy, and RMSE as an index of precision. Median was used as the data displayed a nonnormal distribution. All analyses were performed using R (The R Foundation) [25].



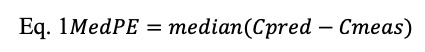





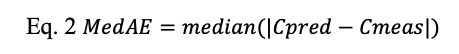





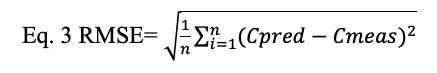



### Funding

This research was internally funded and received no specific grant from any funding agency in the public, commercial, or not-for-profit sectors.

## Results

A total of 93 patient charts were reviewed; 41 charts were excluded for analysis. The main reason for exclusion was the patients being on renal replacement therapy. Twenty-five cases were excluded as they were on renal replacement therapy; 2 cases were excluded due to being pediatric; 2 patients were excluded for model fit; and 12 patients had to be excluded where we were unable to reliably input the patient dose parameters (staggered frequency of dose administration or multiple dosing changes; subsequent vancomycin concentration did not correspond with inputted dose). A total of 104 vancomycin concentrations were assessed. The remaining 52 patients were included in the final analysis. This included 24 (46.2%) female and 28 (53.8%) male participants; age range 24-90 years, median age 63 (SD 14) years; median weight 81.2 kg, weight range 43.6-131.7 (SD 20.8) kg; median BMI 30.7 kg/m^2^, BMI range 16-54.1 (SD 8.6) kg/m^2^. Moreover, 15 (28.8% of the 52 patients were admitted to the intensive care unit.

**Figure 2:**
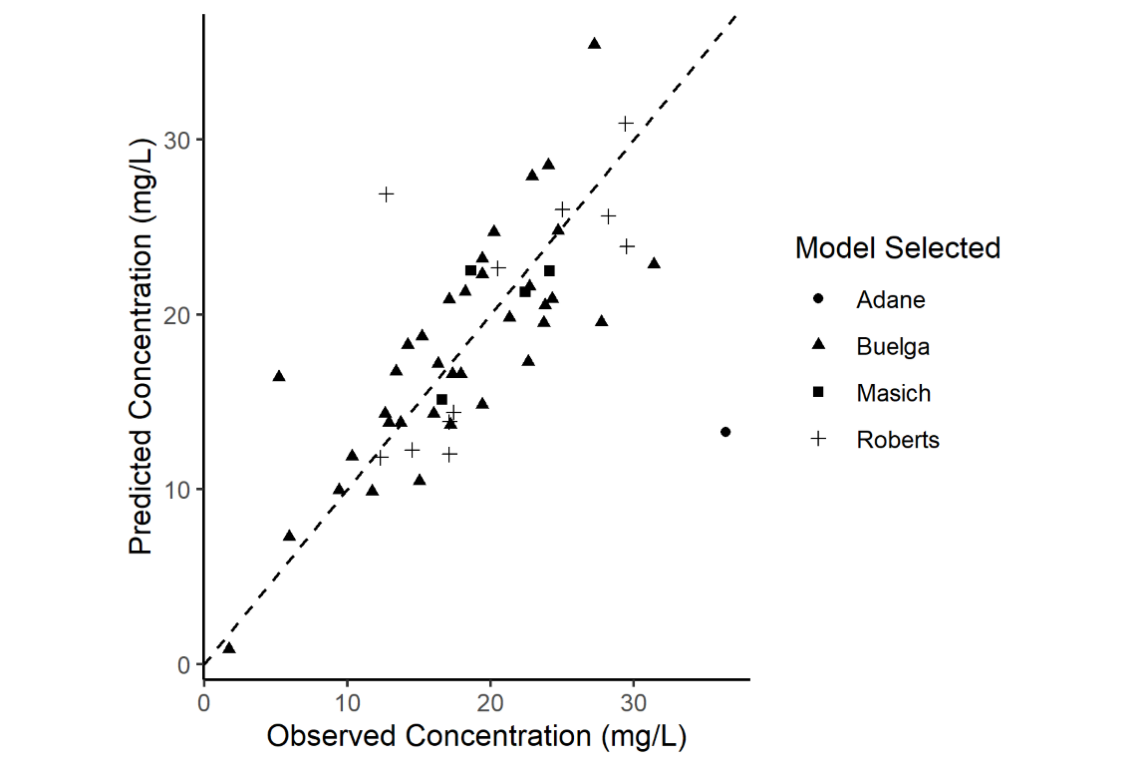
Observed versus predicted vancomycin concentration and an a priori model selected (N=52 from the following models: Adane, n=1; Buelga, n=36; Masich, n=4; and Roberts, n=11).

The median measured concentration was 18.05 (SD 6.83) ug/mL versus the median of the predicted concentration 18.49 (SD 6.40) ug/mL. Using Bayesian estimation, MedPE (bias) was –0.600 ug/mL (IQR –3.06, 2.95), the MedAE (accuracy) was 3.05 ug/mL (IQR 1.44, 4.50), and the RMSE (precision) was 5.34. These results were derived from the following a priori models: Adane et al [11] (n=1); Buelga et al [12] (n=36); Masich et al [14] (n=4); and Roberts et al [13] (n=11; Figure 2). Of note, there were 2 outliers noted in the analysis. One patient was morbidly obese, and the other critically ill. These 2 patients were fit to the Adane et al [11] and Roberts et al [13] a priori models. These outliers were included in the analysis as we could not identify a specific reason for these prediction errors other than their underlying conditions. Performing the analysis excluding these outliers improves median accuracy and precision, with the median bias being largely unchanged. Excluding these outliers, the MedPE was –0.6 ug/mL (IQR –2.9, 2.75), MedAE was 2.95 ug/mL (IQR 1.40, 4.16), and RMSE was 3.86. As other publications have previously reported mean prediction error and mean absolute prediction error [26], we have also included these results. Mean prediction error was –3.13 ug/mL, and mean absolute prediction error was 3.69 ug/mL.

Table 1 displays the bias, accuracy, and RMSE of the individual models. Interpretation of these values is difficult due to the small sample size of some of the patients included in the models.

**Table 1:** App performance of individual pharmacokinetic models.

PK^a^ model	Unique patients, n (%)	Median prediction error (mcg/mL)	Median absolute error (mcg/mL)	Root mean square error
Adane et al [11]	1 (1.9)	–23.14	23.14	23.14
Buelga et al [12]	36 (69.2)	0.34	3.33	4.18
Masich et al [14]	4 (7.7)	–1.29	1.54	2.30
Roberts et al [13]	11 (21.2)	–2.26	2.59	5.20

^a^PK: pharmacokinetic.

## Discussion

### Principal Results

We created a free and open-source Bayesian vancomycin dosing app. No statistical or technical knowledge of Bayesian methods are required to use this app. We focused on making this app easy to use for clinicians, with an ability to explore treatment adjustments.

Vancomycin concentrations in second blood samples were predicted by Bayesian analysis and were compared with measured concentrations to assess the app and PK model accuracy of predictions. Our data represent a small sample size of patients in the Durham region of Ontario. Our total patient sample of 52 patients is small. Hiraki et al [26], Turner et al [27], and Nunn et al [28] all used similar sample sizes in their analysis of Bayesian vancomycin dosing software and PK models.

### Comparisons With Prior Work

We reviewed the findings of Hiraki et al [26], Turner et al [27], and Nunn et al [28]. We chose to use the methods described by Sheiner and Beal [24] to report our predictive accuracy. Hiraki et al [26] did the same; however, other publications chose alternate approaches to reporting predictive accuracy [27,28]. When comparing our prediction accuracy versus those reported by Hiraki et al [26], we showed similar bias and accuracy. However, our overall results were not as precise as the results shared by Hiraki et al [26] (RMSE 5.34 vs 1.74). We attribute this to the 2 outliers within our data set, and as such, these patients may not be ideal candidates for the a priori model selected. Additionally, our data set included a more heterogeneous population of both critically ill and on the general medicine wards, who were being treated with vancomycin for multiple reasons. The population studied by Hiraki et al [26] was limited to hospitalized patients receiving treatment for methicillin-resistant *S. aureus* infections whose renal function was very stable. This could also explain the difference in precision.

Interpretation and performance of the individual PK model accuracy and bias in our paper is limited due to the small sample but are included for review (Table 1). Only 1 patient in the validation data set was fit to the Adane et al [11] model, and 4 patients to the Masich et al [14] model. The predicted result of the single patient fit to the Adane et at [11] model and the one fit to the Roberts et al [13] model produced predicted concentrations that were outliers. The Adane et al [11] model is reserved for patients with a BMI greater than 40 kg/m^2^ and body weight over 120 kg, while the Robers et al [13] model is reserved for the critically ill. Although there were only 2 data points, this highlights the difficulty in dosing these patients. Based on the small number of patients we enrolled during the time frame of this study, it is unlikely that we will be able to obtain a sufficient sample size to evaluate these models individually.

### Limitations

We acknowledge that there are limitations of this app. First, it remains to be determined which a priori PK model is optimal for dosing, since many published vancomycin PK models exist, and choosing the appropriate PK model remains a challenge. It may be unlikely that the wide individual patient PK variability can be captured by a single a priori model. Therefore, it is of utmost importance that the patient be appropriate for the a priori model selected. We chose a one-compartment pharmacokinetic model with individual models for obesity and critical illness. During the development of the app, it appears that a more simplified broadly supported two-compartment model by Goti et al [29] may be more appropriate [30,31].

As trough-based vancomycin dosing was performed at Lakeridge, we are unable evaluate the app’s AUC predictions. However, providing this app free of charge for all users allows the potential for further evaluation of this functionality.

The app is not designed to be used in patients receiving renal replacement therapy. Many patients were excluded from the evaluation as they were receiving vancomycin while on renal replacement therapy.

This app was not intended to replace or override the judgement of a clinician. The app relies on accurate user inputs. There are a few checks in place to ensure appropriate user input and model selection (eg, hard limits on input parameters and goodness of fit with the model), but we acknowledge that all possible scenarios or edge cases cannot be addressed. In the development of the app, we attempted to balance user experience and ease of use versus added complexity and customization of the app and a priori PK models. As suggested earlier, a potential solution to this juxtaposition is a more generally applied model similar to that presented by Goti et al [29], or, if suggested, a more customized user-selected model approach or “expert mode.”

### Future Directions

Bayesian dosing of vancomycin still in its infancy. Vancocalc is available with no restriction. The creation of this free, open-source Bayesian vancomycin dosing app permits this dosing approach to be further explored.

The value of free, open-source software is unquantifiable. Software such as Linux and Python have positively impacted many. We are hopeful that the creation of this app can also have a positive impact and add to the body of work that has already been conducted [16,17].

With increased use and validation of Bayesian dosing apps, it is possible that improved PK models for specific patient populations will be developed. Additionally, larger adoption may eventually permit improved algorithms incorporating other techniques such as machine learning. By open sourcing the app, it can be modified, updated, and improved upon or used as a framework for others to build upon.

Our goal is to share this proof-of-concept app to allow greater awareness and democratization of the Bayesian monitoring approach. The pharmacokinetics of vancomycin are complex. With greater use and awareness of the app and experience from field experts, this will lead to further collaboration and improvement of the app for functionality and vancomycin TDM.

### Conclusions

In this paper, we presented a vancomycin dosing app, VancoCalc, including a small evaluation using a real-world retrospective data set. This app leverages previously published research on Bayesian inference calculators. By offering this free, open-source app, further prospective validation could be implemented in the near future. We encourage exploration of the app and collaboration or suggestions for improvement.

## Abbreviations

AUC: area under the curve
Cl: clearance
IV: intravenous
MedAE: median absolute error
MedPE: median prediction error
PK: pharmacokinetic
RMSE: root mean squared error
TDM: therapeutic drug monitoring
VD: volume of distribution
